# Comparison of timing of relapse in dogs with nonassociative immune‐mediated hemolytic anemia, thrombocytopenia, or polyarthritis

**DOI:** 10.1111/jvim.17004

**Published:** 2024-02-02

**Authors:** Richard Sparrow, James W. Swann, Barbara Glanemann

**Affiliations:** ^1^ Department of Clinical Science and Services Royal Veterinary College Hatfield UK; ^2^ Columbia Stem Cell Initiative Columbia University New York New York USA

**Keywords:** autoimmune, canine, idiopathic, vaccination

## Abstract

**Background:**

Relapse is a clinical concern in dogs diagnosed with immune‐mediated hemolytic anemia (IMHA), thrombocytopenia (ITP), or polyarthritis (IMPA). The average time to relapse is unknown, and evidence that vaccination is associated with disease relapse is lacking.

**Hypothesis/Objectives:**

Compare the incidence of relapse in groups of dogs with IMHA, ITP, or IMPA over a 24‐month period after diagnosis and compare proportions of dogs that received vaccines in those dogs that did and did not relapse.

**Animals:**

One hundred sixty client‐owned dogs (73 with IMHA, 55 with ITP, 32 with IMPA).

**Methods:**

Medical records of dogs were reviewed with the goal of following cases for a minimum of 2 years. Incidence of relapse was calculated for each disease, and relapse rates in dogs that were or were not vaccinated after diagnosis were compared.

**Results:**

Relapse rates at 12 months differed significantly among disease groups (*P* = .02), with a higher rate for IMPA (35%) compared to IMHA (11%) or ITP (11%). Relapse rate at 24 months was 41% for IMPA, 18% for IMHA, and 23% for ITP. Ninety percent of IMPA relapses occurred in the first 12 months after diagnosis, compared with 56% for IMHA and 50% for ITP. Vaccine administration after diagnosis was not associated with relapse (*P* = .78).

**Conclusions and Clinical Importance:**

Risk of disease relapse in IMPA is highest in the first year after diagnosis, with a higher relapse rate compared with IMHA and ITP. The role of vaccination in disease relapse remains unclear.

**Abbreviations**
IMHA: immune‐mediated hemolytic anemia
IMPA: immune‐mediated polyarthritis
ITP: immune thrombocytopenia

## 1 INTRODUCTION

Immune‐mediated hemolytic anemia (IMHA), immune thrombocytopenia (ITP), and immune‐mediated polyarthritis (IMPA) are 3 of the most common immune‐mediated diseases of dogs caused by loss of immune tolerance to self‐antigens.^1^, ^2^, ^3^ Termed primary or nonassociative in the absence of a detectable underlying cause,^4^ this group of conditions causes considerable mortality and morbidity despite advances in supportive care and immunosuppressive treatment.^5^, ^6^, ^7^

Relapse of clinical disease is a possible feature of all immune‐mediated conditions and is a concern for both clinicians and owners because relapses may be associated with increased morbidity or mortality. Reported prevalence of relapse ranges between 11% and 24%^5^, ^8^, ^9^, ^10^, ^11^ for IMHA, 9% and 39%^6^, ^12^, ^13^, ^14^ for ITP, and 20% and 48%^3^, ^15^, ^16^ for IMPA. However, it remains unclear whether or not the likelihood of relapse decreases with time after diagnosis, and whether or not the timing of relapse after diagnosis is similar among all 3 diseases. Consequently, it is difficult for clinicians to advise owners about the likelihood of relapse, the most likely timing of relapse, and the likely duration of immunosuppressive treatment. Collectively, this uncertainty creates a clinical need for accurate information about the timing of relapses.

Various potential trigger factors have been associated with initial onset of immune‐mediated diseases, including infectious pathogens, vaccination, drugs, toxins, inflammatory diseases, and neoplasia.^4^ However, apart from some infectious agents, evidence supporting a causal relationship is lacking in most cases. Temporal relationships between vaccination and initial onset of immune‐mediated diseases have been investigated^17^, ^18^, ^19^ but, to our knowledge, no evidence suggests that vaccination will induce relapse in a dog in remission from an immune‐mediated disease. Association between vaccination and relapse among dogs previously diagnosed with IMHA is unclear.^20^ Regardless, 42.7% (70/164, 95% confidence interval [CI], 33.3‐53.9) of veterinarians in the United Kingdom would elect to withhold vaccination of dogs previously diagnosed with IMHA,^21^ suggesting relapse is a major clinical concern. Nevertheless, it remains unclear whether relapse is more likely in those dogs with immune‐mediated diseases that go on to receive vaccination compared to those from which vaccinations are withheld.

Our primary aim was to compare the incidence of relapse in groups of dogs diagnosed with IMHA, ITP, or IMPA with the aim of following dogs for 24 months after diagnosis, and to describe the time period between diagnosis and relapse. Considering the ranges of previously reported relapse rates, we hypothesized that there would be no significant difference in rate of relapse among the different diseases. A second aim was to determine the rate of vaccination after diagnosis, and to compare rates of relapse among dogs that did or did not receive vaccinations.

## 2 MATERIALS AND METHODS

### 2.1 Study design

A retrospective cohort study was performed to compare relapse rates among dogs with IMHA, ITP, or IMPA, and a retrospective case control study was conducted to compare rates of relapse among dogs with immune‐mediated diseases that were or were not subsequently vaccinated. In our study, the term “nonassociative” is used to describe dogs with IMHA as previously suggested,^4^ and also is applied to dogs with primary ITP or IMPA. Ethical approval for the study was granted by the Royal Veterinary College Social Sciences Ethical Review Board (URN SR2019‐0378).

### 2.2 Data collection

The electronic medical record system of a referral teaching hospital was searched during a 2‐year period (January 1, 2015 through December 31, 2017) for dogs presented with IMHA, ITP, or IMPA using the following search terms: immune‐mediated hemolytic anemia, IMHA, AIHA, hemolytic anemia, immune‐mediated thrombocytopenia, IMTP, ITP, immune‐mediated polyarthritis, IMPA, polyarthritis, polyarthropathy. Records were reviewed for suitability for inclusion based on diagnostic criteria for each disease.

### 2.3 Inclusion and exclusion criteria

Dogs were included in the IMHA group if they satisfied the diagnostic criteria outlined in the American College of Veterinary Internal Medicine consensus statement on the diagnosis of IMHA.^4^ These included all of the following:

- Anemia, with packed cell volume (PCV) <35%.
- One or more signs of immune‐mediated red blood cell destruction, including positive saline agglutination test, or presence of spherocytosis, or positive direct antiglobulin test (DAT).
- At least 1 indicator of hemolysis, including hyperbilirubinemia, or bilirubinuria, or hemoglobinuria.

Inclusion in the ITP group required:

- Thrombocytopenia (platelet concentration <50 000/μL), confirmed by fresh blood smear examination, with no identifiable alternative cause of thrombocytopenia after diagnostic evaluation.

Dogs with IMPA were included if there was:

- Consistent history of ≥1 compatible clinical signs, including lameness, stiffness, reluctance to walk, pyrexia, or joint effusions.
- Cytological evidence of nonseptic neutrophilic inflammation (>30% neutrophils among nucleated cells) upon arthrocentesis of ≥3 joints.

In all 3 groups, dogs were included only if they survived beyond the point of discharge. Additionally, all dogs were required to have screening for possible associative conditions with a minimum database of:

- CBC with blood smear review by a board‐certified clinical pathologist.
- Serum biochemical profile with no abnormalities other than those consistent with the immune‐mediated disease (eg, hyperbilirubinemia in dogs with IMHA).
- Abdominal imaging with no findings that were considered to be possible causative factors for immune‐mediated disease.
- Infectious disease testing if the medical records indicated that there was a history of travel outside of the United Kingdom.

Data regarding additional diagnostic testing, including thoracic imaging, urinalysis, and screening for infectious diseases, were collected where available but this information was not essential for inclusion. Dogs were excluded if medical records were incomplete, or if review of medical records identified evidence of a potential associative condition. Dogs previously diagnosed with IMHA, ITP, or IMPA that presented during the study period with a relapse of disease were excluded. However, these dogs were included in the case control study comparing rates of relapse in dogs that were or were not vaccinated after initial diagnosis.

Data collected for all dogs included signalment, clinicopathological data, and details of immunosuppressive treatment. Information regarding clinical progression after diagnosis was obtained from review of hospital records and by contacting referring veterinarians by telephone or email, aiming to obtain at least 24 months of follow‐up for every case. This included follow‐up time period, characteristics of initial immunosuppressive treatment and whether it was terminated (owing to disease remission), date and timing of relapse(s), status of immunosuppressive treatment at the time of relapse, record of vaccination after diagnosis, and whether the dog was receiving immunosuppressive drugs at the time of vaccination.

### 2.4 Remission and relapse

Remission of disease was defined as follows:

- IMHA: PCV ≥30% and clinical signs resolved.
- ITP: platelet concentration ≥150 000/μL and clinical signs resolved.
- IMPA: clinical signs resolved.

Additionally, in all disease groups, reduction of immunosuppressive treatment was considered an essential characteristic of remission because it indicated that the attending clinician believed the dog was in remission at the time of clinical evaluation.

Relapses were defined as follows:

- IMHA: decrease in PCV by >5% from previous result (eg, decrease from 30% to 24%), after discharge and at least 2 weeks after the last blood transfusion.
- ITP: decrease in platelet concentration to <150 000/μL after previously being ≥150 000/μL, confirmed by fresh blood smear examination by a board‐certified veterinary clinical pathologist or hematology technician.
- IMPA: recurrence of ≥1 compatible clinical sign(s) after previous resolution.

Additionally, in all disease groups, intensification of immunosuppressive treatment was considered an essential characteristic of relapse because it indicated that the attending clinician believed relapse was occurring at the time of clinical evaluation. This included any of the following: reinstitution of immunosuppressive treatment if previously stopped, increase in immunosuppressive drug dose, exchange of second immunosuppressive agent for another drug, or addition of another immunosuppressive agent. Temporal association between relapse and vaccination was considered possible if a relapse event occurred within 30 days of vaccine administration.

### 2.5 Statistical analysis

Analysis was performed using a statistical software package (SPSS Statistics, version 28; IBM). Normality of continuous variables was assessed using Shapiro‐Wilks tests; central tendency in normally distributed variables is summarized with mean and SD and in nonnormally distributed variables with median and interquartile range (IQR). Kaplan‐Meier product limit estimates with log‐rank test were used to compare timing of relapses among the different disease groups. Cases were censored from the analysis at the point of loss to follow‐up. Chi‐squared tests were used to compare rates of relapse among diseases at 12 and 24 months after diagnosis, with post hoc z tests for pairwise comparisons where significant associations were detected. Chi‐squared tests or Fisher's exact tests also were used to compare relapse rates in dogs with immune‐mediated diseases that were or were not subsequently vaccinated, and to compare use of second immunosuppressive agents at discharge among dogs that did and did not relapse, depending on the number of cases per cell. Statistical significance was defined as a *P* value <.05.

## 3 RESULTS

### 3.1 Immune‐mediated hemolytic anemia

#### 3.1.1 Clinical characteristics

The electronic medical records search identified 116 dogs with IMHA, of which 43 were excluded, leaving 73 dogs for inclusion in the study. Reasons for exclusion were: initial diagnosis not within the study period (n = 6), no CBC (n = 1), no serum biochemical profile (n = 1), no markers of immune‐mediated destruction (n = 6), no markers of hemolysis (n = 3), no abdominal imaging (n = 2), suspected associative disease (n = 5), nonsurvival to discharge (n = 19; Supplementary Figure 1). Group demographics are summarized in Supplementary Table 1. The most common breeds in the IMHA group were: mixed breed (n = 8, 11%), English Cocker spaniel (8, 11%), Springer spaniel (7, 10%), and Jack Russell terrier (7, 10%), with an additional 43 dogs of 30 other breeds. Features of diagnostic testing and clinicopathological data are presented in Supplementary Tables 2 and 3.

#### 3.1.2 Relapse

**FIGURE 1:**
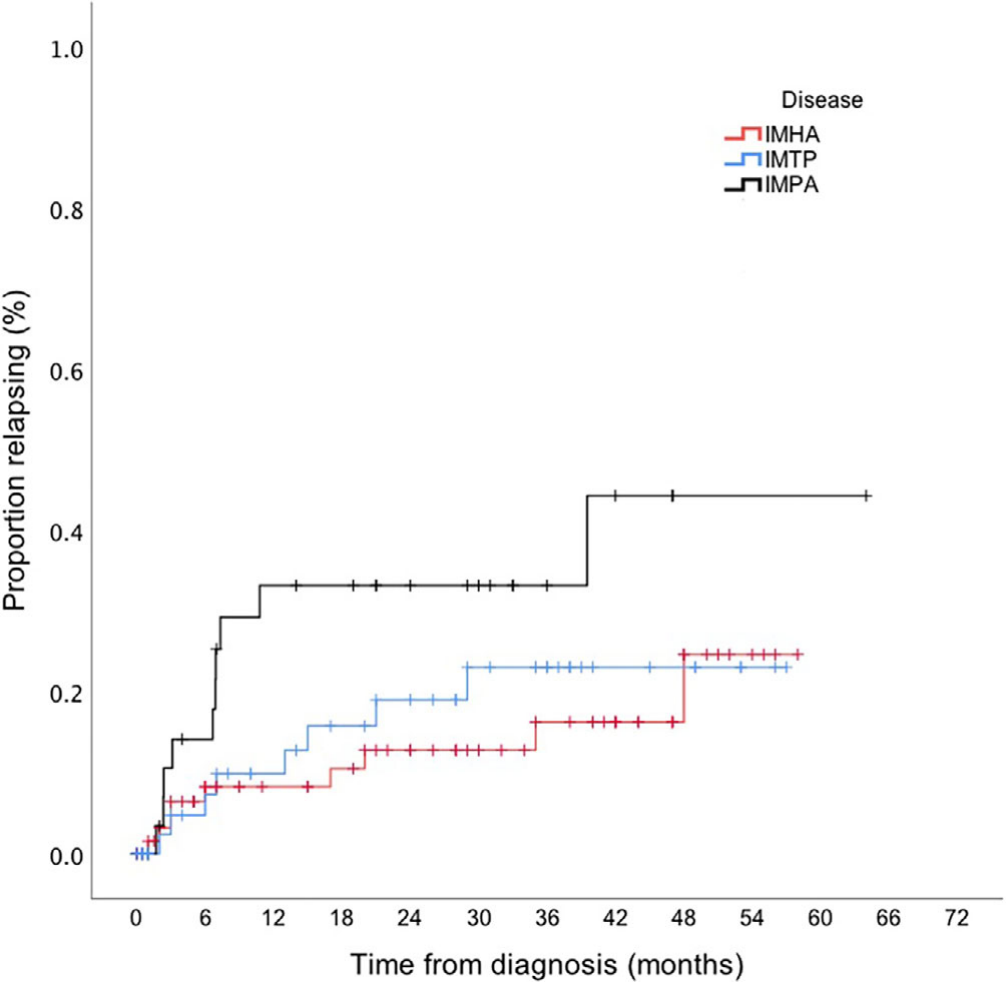
Kaplan Meier curve comparing incidence of relapse between dogs diagnosed with IMHA, ITP and IMPA. Censor marks (+) indicates point of loss to follow up in a dog that did not relapse.

**FIGURE 2:**
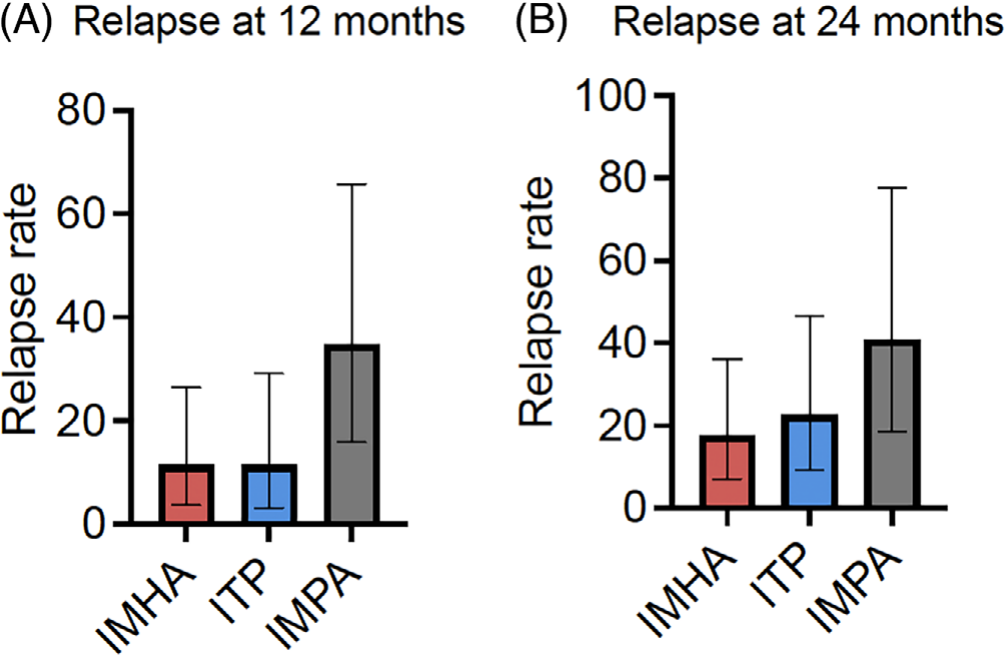
Bar charts comparing relapse rates among dogs with IMHA, ITP and IMPA. (A) relapse rate among evaluable dogs <12 months after diagnosis. (B) Relapse rate among evaluable dogs <24 months after diagnosis.

Five of 73 (7%) dogs diagnosed with IMHA were presented with relapse of previously diagnosed IMHA, and these were excluded from analysis of relapse incidence, leaving 68 dogs. Nine dogs diagnosed with IMHA experienced a relapse, with 5 (56%) occurring within 12 months of diagnosis and an additional 2 occurring between 12 and 24 months (Figure 1). Two (22%) dogs experienced a relapse >24 months after their initial diagnosis: at 35 months and at 48 months. Relapse rate was therefore 5/44 (11%; 95% CI, 3.7‐26.5) and 7/40 (18%; 95% CI, 7.0‐36.1) for cases with complete follow‐up to 12 and 24 months, respectively (Figure 2, Supplementary Table 4). Median time to relapse was 6 months (interquartile range [IQR], 2.5‐27.5). Six of the 9 relapsing dogs (67%) were still receiving immunosuppressive treatment at the time of relapse. Two dogs developed a new immune‐mediated disease during the follow‐up period; 1 dog developed ITP 21 months after diagnosis of IMHA, and the other dog developed immune‐mediated dermatopathy at 15 months. Both dogs were receiving immunosuppressive drugs at the time they developed the new immune‐mediated disease.

Eleven of 68 (16%) dogs were lost to follow‐up and 17 (25%) died or were euthanized <24 months after diagnosis, leaving 44 dogs evaluable at 12 months after diagnosis, and 40 dogs at 24 months after diagnosis. Stated reasons for euthanasia included: poor response to treatment (n = 2), medication adverse effects or poor quality of life (n = 2), cost of treatment (n = 2), paraparesis (n = 1), suspected pancreatitis (n = 1), pneumonia with multiple organ dysfunction syndrome (n = 1), hemoabdomen (n = 1), pneumoperitoneum (n = 1), and unknown (n = 2). The cause of death in 4 dogs that died was unknown. Five dogs died or were euthanized owing to relapse or development of a new immune‐mediated disease.

#### 3.1.3 Treatment

Characteristics of the initial treatment protocol are summarized in Supplementary Table 5. All dogs were receiving prednisolone at the time of discharge, at a median dosage of 2.2 mg/kg/day (IQR, 1.97‐2.98). The first prednisolone dose reduction occurred a median of 22 days (IQR, 17.3‐27.0; n = 52) after diagnosis, and the second prednisolone dose reduction occurred a median of 21 days (IQR, 14.8‐29.0; n = 42) after the first. The prednisolone dosage was decreased to a median of 1.6 mg/kg/day (IQR, 1.47‐2.04) at the first reduction, and to a median 1.1 mg/kg/day (IQR, 0.96‐1.30) at the second. Median duration of prednisolone treatment was 5 months (IQR, 3.9‐6.8) for 45 dogs with sufficient follow‐up data to establish the termination of treatment. Median duration of second agent treatment was 6 months (IQR, 5.2‐7.8; n = 34). Thirty‐four of 40 (85%) dogs with 24 months follow‐up were discharged receiving a second immunosuppressive agent, including 67% (6/9) of dogs that relapsed and 90% (28/31) of dogs that did not relapse. Immunosuppressive drugs ultimately were discontinued in 76% (54/71) of dogs.

### 3.2 Immune thrombocytopenia

#### 3.2.1 Clinical characteristics

The electronic medical records search identified 79 dogs with ITP, of which 13 were excluded, leaving 55 dogs for inclusion in the study. Reasons for exclusion were: initial diagnosis not within the study period (n = 2), platelet concentration >50 000/μL (n = 3), no abdominal imaging (n = 4), suspected associative disease (n = 4), nonsurvival to discharge (n = 11; Supplementary Figure 2). Group characteristics are summarized in Supplementary Table 1. The most common breeds were Cocker spaniel (n = 9, 16%), mixed breed (8, 15%), English Springer spaniel (5, 9%), Labrador retriever (4, 7%), with an additional 29 dogs of 25 other breeds. Features of diagnostic testing and clinicopathological data are shown in Supplementary Tables 2 and 3.

#### 3.2.2 Relapse

Two of 55 (4%) dogs diagnosed with ITP were presented with a relapse of historical disease and were excluded from analysis of relapse incidence, leaving 53 dogs. Eight dogs diagnosed with ITP experienced a relapse, with 4 relapses occurring within 12 months and 3 relapses occurring between 12 and 24 months after diagnosis (Figure 2, Supplementary Table 4). One dog experienced a relapse only after >24 months from initial diagnosis, occurring at 29 months. Median time to a relapse was 10 months (IQR, 3.75‐19.5). Relapse rate was 4/35 (11%; 95% CI, 3.1‐29.2) and 7/31 (23%; 95% CI, 9.1‐46.5) for cases evaluable at 12 and 24 months, respectively. Half of the dogs relapsed while still receiving immunosuppressive treatment.

Nine (17%) dogs were lost to follow up <24 months after diagnosis. Fourteen (26%) dogs died or were euthanized within 24 months after diagnosis, and 1 of these dogs relapsed at 11 months. Therefore, 35 dogs were evaluable at 12 months after diagnosis, and 31 dogs at 24 months. Stated reasons for euthanasia included: proven or suspected sepsis (n = 3), pancreatitis (n = 2), septic arthritis (n = 1), steroid‐related adverse effects (n = 1), arthritic disease (n = 1), and 1 dog was euthanized shortly after discharge owing to suspected development of concurrent IMHA. Cause of death was unknown in 3 of 4 dogs that died and was attributed to suspected thromboembolic disease in the remaining dog. All dogs that relapsed had follow‐up data for at least 24 months after diagnosis, other than 1 dog that was euthanized owing to relapse.

#### 3.2.3 Treatment

All dogs were being treated with prednisolone at the time of discharge, at a median dosage of 2.19 mg/kg/day (IQR, 1.92‐2.55). The first prednisolone dose reduction occurred a median of 28 days (IQR, 17.5‐37.0; n = 36) after diagnosis, and the second reduction occurred a median of 24 days (IQR, 14.8‐30.3; n = 30) after the first. The prednisolone dosage was decreased to median of 1.67 mg/kg/day (IQR, 1.50‐1.90) at the first reduction, and to 1.12 mg/kg/day (IQR, 0.91‐1.30) at the second. Median duration of prednisolone treatment was 6 months (IQR, 4.4‐8.4) for 30 dogs with sufficient follow‐up data to establish termination of treatment. Twenty‐two of 31 dogs with 24 months follow‐up were discharged receiving a second immunosuppressive drug, including 75% (6/8) of dogs that relapsed and 67% (16/24) of dogs that did not relapse. Median duration of second agent treatment was 7 months (IQR, 5.0‐8.6; n = 16). Immunosuppressive treatments ultimately were discontinued in 33% (17/52) of dogs.

### 3.3 Immune‐mediated polyarthritis

#### 3.3.1 Clinical characteristics

The electronic medical records search identified 57 dogs with IMPA, of which 25 were excluded, leaving 32 dogs for inclusion in the study. Reasons for exclusion were: initial diagnosis not within the study period (n = 3), arthrocentesis performed on <3 joints, neutrophilic inflammation documented in <3 joints, no abdominal imaging (n = 6), confirmed leishmaniasis (n = 1), concurrent steroid‐responsive meningitis‐arteritis (n = 1) and suspected associative disease (n = 4; Supplementary Figure 3). Group characteristics are summarized in Supplementary Table 1. The most common breeds were English Cocker spaniel (n = 6, 19%), mixed breed (4, 13%), English Springer spaniel (3, 10%), standard Poodle (2, 6%), with 17 dogs of 17 other breeds. Lameness and joint effusion were the most common clinical signs, each occurring in 25 (78%) dogs, followed by pyrexia (n = 22, 69%), reluctance to walk (16, 50%), and stiffness (15, 47%). Features of diagnostic testing are presented in Supplementary Table 2.

#### 3.3.2 Relapse

Ten dogs with IMPA experienced a relapse: 90% of these relapses occurred within the first 12 months after diagnosis, with 1 additional dog relapsing at 40 months (Figure 1). Median time to relapse was 7 months (IQR, 2.4‐8.2). Relapse rate was therefore 9/26 (35%; 95% CI, 15.8‐65.7) at 12 months and 9/22 (41%; 95% CI, 18.7‐77.7) at 24 months (Figure 2, Supplementary Table 4). Six (60%) dogs that relapsed did so after ceasing immunosuppressive treatment, whereas 4 (40%) still were receiving treatment at the time of relapse. One of these 4 dogs experienced relapses both when still receiving and after discontinuation of immunosuppressive treatment, at different time points. Five of 10 dogs that relapsed experienced >1 relapse: 4 dogs had 2 relapses and 1 dog had 3 relapses. Second relapses occurred a median of 3 months (IQR, 2.4‐8.2) after the first. The dog that relapsed 3 times experienced the third relapse 12 months after the first, and 7 months after the second.

Five (16%) dogs were lost to follow‐up <24 months after diagnosis. In addition, 6 (27%) dogs died or were euthanized <24 months after diagnosis, and 1 of these dogs relapsed at 2 months. Therefore, 26 dogs were evaluable at 12 months after diagnosis, and 22 dogs at 24 months. Stated reasons for euthanasia included aggression (n = 2), seizures (n = 1), and suspected pancreatitis and thromboembolism (n = 1). One dog died at home with unknown cause. One dog with IMPA relapsed at 7 months and had follow‐up data available up to 21 months after diagnosis.

#### 3.3.3 Treatment

All dogs were being treated with prednisolone at the time of discharge, at a median dosage of 2.42 mg/kg/day (IQR, 1.88‐3.00). The first prednisolone dose reduction occurred at a median of 20 days (IQR, 15.0‐24.8; n = 24) after diagnosis, and the second reduction occurred at a median of 21 days (IQR, 12.5‐32.3; n = 19) after the first. The prednisolone dosage was decreased to a median of 1.56 mg/kg/day (IQR, 1.41‐1.92) at the first reduction, and to 1.11 mg/kg/day (IQR, 0.95‐1.32) at the second. Median duration of prednisolone treatment was 5 months (IQR, 3.3‐7.1) for 19 dogs with sufficient follow‐up data to establish termination of treatment. Eighteen of 32 dogs (56%) were discharged receiving prednisolone in combination with a second immunosuppressive agent (Supplementary Table 5). Median duration of second agent treatment was 8 months (IQR, 3.3‐13.6; n = 6). Thirteen of 22 (59.1%) dogs were discharged receiving a second immunosuppressive drug, including 70% (7/10) of dogs that relapsed and 50% (6/12) of dogs that did not relapse. Immunosuppressive treatments ultimately were discontinued in 69% (20/29) of dogs.

### 3.4 Comparison of relapse rates among IMHA, ITP, and IMPA

The proportion of dogs experiencing a relapse by 12 months after diagnosis differed significantly among disease groups (*X* ^*2*^ = 7.427, *P* = .02). Although a higher relapse rate was observed among dogs with IMPA compared to dogs with either IMHA or ITP, post hoc z scores did not indicate a significant difference in these proportions. We did not observe any difference in relapse rate among disease groups by 24 months after diagnosis (*X* ^*2*^ = 4.294, *P* = .12).

### 3.5 Vaccination

Across all disease groups, 114 of 160 (71%) dogs had follow‐up data available to evaluate vaccination after diagnosis. Overall, 56/114 (49%) dogs received a vaccine after their initial diagnosis, including 29/51 (57%) dogs with IMHA, 14/37 (38%) with ITP, and 13/26 (50%) with IMPA. No dog relapsed <30 days after vaccination, regardless of whether or not they were concurrently receiving immunosuppressive drugs. In summary, 21 of 29 dogs with IMHA were vaccinated when off immunosuppressive drugs, as were 8 of 14 dogs with ITP and 8 of 13 dogs with IMPA. Sixteen of 35 (46%) dogs that relapsed, or presented during the study period with a relapse of disease that was previously in remission, were vaccinated after their initial diagnosis. This number included 8 dogs with IMHA, 3 dogs with ITP, and 5 dogs with IMPA. Of these dogs, 6 with IMHA, 2 with ITP and 3 with IMPA were vaccinated when off immunosuppressive drugs. Vaccination was not suspected to be a trigger for relapse in any of these dogs because all relapses occurred >30 days after vaccine administration. Initial diagnosis also was not temporally associated with vaccination in the 16 dogs with a previous history of immune‐mediated disease: 15 dogs were not vaccinated <30 days before diagnosis, but the date of most recent vaccination was unknown in 1 dog. No difference was found in the proportion of relapsing dogs that received a vaccine compared to the proportion of relapsing dogs that did not receive a vaccine (*X* ^*2*^ = .076, *P* = .78). One dog that developed an immune‐mediated dermatopathy after remission of IMHA was vaccinated after the diagnosis of IMHA, but the vaccine was administered while the dog was still receiving immunosuppressive drugs, 11 months before the onset of the immune‐mediated dermatopathy.

## 4 DISCUSSION

In our study of dogs diagnosed with immune‐mediated diseases, we found that relapse rates differed significantly among dogs with different diseases at 12 months, but no significant difference in relapse rate was found 2 years after diagnosis. Most relapses in dogs with IMPA were observed in the first 12 months after diagnosis, whereas dogs with IMHA and ITP relapsed later. Approximately half of dogs received a vaccine after diagnosis of an immune‐mediated disease, no association was found between vaccination and relapse.

Forty percent of dogs with IMPA relapsed during the follow‐up period, and 90% of these relapses occurred within the first 12 months after diagnosis. These results were similar to those of a previous study, which reported that 7/12 (58%) dogs with IMPA relapsed within 12 months of diagnosis, with 1 additional dog relapsing after a year.^3^ We therefore propose that the first year after diagnosis be considered the highest risk period for relapse in dogs with IMPA. Dogs with IMPA that did not relapse in the first year after diagnosis were unlikely to do so subsequently, suggesting this group has a more favorable long‐term prognosis.

In dogs with ITP or IMHA, a lower proportion of relapses occurred in the first year after diagnosis, with relapses observed in dogs with IMHA in the second, third, and even fifth years after diagnosis. Relapse in dogs with IMHA >2 years after diagnosis is described, with occurrences between 32 and 1757 days after diagnosis (approximately 1‐62 months),^9^ and a separate study indicated that 10/14 dogs with IMHA that relapsed did so >12 months after diagnosis.^11^ We therefore emphasize the importance of awareness, among owners and clinicians, that disease relapses might occur sporadically in dogs with IMHA and ITP, even in those that have been in remission for several years.

Additionally, we observed 2 dogs that developed a new immune‐mediated disease, both >12 months after diagnosis of IMHA. It remains unclear whether these syndromes were attributable to epitope spreading in the original autoimmune response or whether they reflected an underlying predisposition to developing de novo autoimmune responses to diverse self‐antigens. Nevertheless, this observation suggests that clinicians and owners should remain vigilant for development of new immune‐mediated diseases in dogs with a previous history of any immune‐mediated disease.

We observed a higher rate of relapse among dogs with IMPA 12 months after diagnosis, compared with either IMHA or ITP, although pairwise comparisons among disease groups did not identify a significant difference in these proportions, probably owing to the lower number of dogs in the IMPA group. This observation also should be interpreted in the context of low numbers of dogs that experienced disease relapse. If validated in a larger population of dogs, this possible difference could be related to the subjective nature of relapse assessment in dogs with IMPA, which was based on recurrence of clinical signs, without requiring objective measures of assessment in our study. Because no clinical sign is specific for IMPA, this might falsely increase relapse rates if dogs previously diagnosed with IMPA were presented with signs attributable to other diseases, such as orthopedic disease. Repeated arthrocentesis or measurement of C‐reactive protein could have increased confidence that relapse was occurring, but these tests were not performed in most dogs that relapsed in our study. Conversely, monitoring of remission in IMHA and ITP can be assessed objectively using a CBC, making the diagnosis of relapse relatively unambiguous. A further possible explanation for the difference in relapse rate among diseases could be variations in treatment protocols. We did not find a difference in rates of use of combination immunosuppressive treatments between dogs that did or did not relapse in any disease group, but our analysis may be underpowered for this comparison because only small numbers of dogs were treated with prednisolone alone. Different protocols for tapering and duration of immunosuppressive treatment could have an effect on the likelihood of relapse, but we found that key features of the immunosuppressive treatment of dogs with IMPA, such as times to initial dose reductions, total duration of treatment, and initial and tapered prednisolone dosages, were comparable to those in dogs with IMHA and ITP. These findings suggest that variation in immunosuppressive protocols may not explain the higher relapse rate at 12 months for dogs with IMPA, as also suggested by the fact that dogs with IMPA that were treated with second immunosuppressive agents were maintained on those drugs for longer than dogs with ITP or IMHA. Finally, differences in relapse rate could reflect an underlying difference in the nature of the autoimmune response occurring in each disease, or in the sensitivity of these disease processes to conventional immunosuppressive treatment.

We found that approximately half of dogs were vaccinated after diagnosis of an immune‐mediated disease. This observation validates recent questionnaire data, indicating that 42.7% of veterinarians would elect not to vaccinate a dog previously diagnosed with IMHA in the United Kingdom in a simulated clinical scenario.^21^ Regardless, we did not observe an association between vaccination and relapse, although this conclusion is complicated by the fact that many dogs were still receiving immunosuppressive drugs at the time of vaccination, which might mask any recrudescence of disease. We defined a temporal association between vaccination and relapse as a relapse within 30 days of vaccination. An approximately 4‐week period is commonly used to identify temporal associations,^6^, ^18^ but this timeframe is arbitrary. Therefore, it is possible that a 30‐day period could underestimate associations between vaccination and relapse. Additionally, differences in vaccines types, including epitopes and adjuvants used, and the individual immune response, may play a role in the likelihood of disease relapse. Regardless, although the decision to vaccinate individual dogs after diagnosis of an immune‐mediated disease still should be guided by consideration of risk‐benefit ratio, we suggest the risk of vaccine‐associated relapse is low.

Our study had some additional limitations. Some dogs were lost to follow‐up, with potential for bias if those dogs that had a poorer response to treatment or experienced relapse were more likely to be presented for reexamination. We also cannot rule out the possibility that some cases were lost to follow‐up owing to death or euthanasia caused by a relapse of their original disease. There was no standardization of initial treatment protocols in our study, nor was there any uniform approach to tapering immunosuppressive medications over time, which could influence the likelihood of relapse. Our study was based on a referral hospital sample of dogs, and may not be representative of the wider population presenting in primary care practice. Some dogs did not have urinalysis, fecal analysis, thoracic imaging, or infectious disease screening performed. Although unlikely, occult associative disease therefore cannot be completely excluded, and if present might have affected treatment response or likelihood of relapse in some dogs. The majority of dogs underwent diagnostic imaging of both the thorax and abdomen, but thoracic imaging was not performed in a small number of dogs. Recent studies of dogs with IMHA suggest this omission is unlikely to have affected our results, with thoracic imaging being a low yield screening test that is unlikely to identify abnormalities or an underlying trigger factor.^22^, ^23^, ^24^ Not all dogs had infectious disease screening performed, although doing so was essential if there was a history of travel, and the occurrence of immune‐mediated diseases secondary to infectious agents is rare in the United Kingdom where the study was performed. The mainstay of infectious disease testing in our study was by serological methods, which could also yield false negative results in acute infections. However, dogs with unidentified infectious disease would not be expected to respond well to immunosuppression and survive to discharge, which was an inclusion criterion in our study. Additionally, we assessed long‐term follow‐up of dogs receiving immunosuppressive drugs, and review of the medical records would have likely identified if an unmasking of occult infectious disease occurred in those dogs. Regardless, we cannot exclude the possibility that occult infectious disease could have been present in some dogs and might have affected their response to treatment or the likelihood of relapse. Not all dogs with IMPA had radiographs of the joints to assess for erosive disease, and dogs with erosive IMPA might have poorer responses to treatment. However, erosive IMPA is rare and therefore unlikely to have affected our results. Finally, among dogs with ITP, although the diagnosis was made by board‐certified internists or criticalists, conditions resulting in consumption or sequestration of platelets, and bone marrow disease, cannot be completely excluded in some dogs because screening for coagulopathies and bone marrow cytology was not performed routinely.

In conclusion, we found that relapse in dogs with IMPA was most likely to occur within the first year after diagnosis whereas relapses in dogs with ITP were more evenly distributed between the first and third years after diagnosis, and dogs with IMHA may be at risk of relapse for several years after their initial diagnosis.

## CONFLICT OF INTEREST DECLARATION

Authors declare no conflict of interest.

## OFF‐LABEL ANTIMICROBIAL DECLARATION

Authors declare no off‐label use of antimicrobials.

## INSTITUTIONAL ANIMAL CARE AND USE COMMITTEE (IACUC) OR OTHER APPROVAL DECLARATION

Approved by the Royal Veterinary College Social Sciences Ethical Review Board (URN SR2019‐0378).

## HUMAN ETHICS APPROVAL DECLARATION

Authors declare human ethics approval was not needed for this study.

## Supporting information


**Supplementary Figure 1.** Flow diagram demonstrating reasons for exclusion among 116 dogs identified with IMHA during the study period. *5 dogs presenting with relapse of historical IMHA included in the case‐control study comparing rates of relapse in dogs that were or were not vaccinated after initial diagnosis. IMHA, immune‐mediated hemolytic anemia.
**Supplementary Figure 2.** Flow diagram demonstrating reasons for exclusion among 79 dogs identified with ITP during the study period. *2 dogs presenting with relapse of historical ITP included in the case‐control study comparing rates of relapse in dogs that were or were not vaccinated after initial diagnosis. ITP, immune thrombocytopenia; PLT, platelet concentration.
**Supplementary Figure 3.** Flow diagram demonstrating reasons for exclusion among 57 dogs identified with IMPA during the study period. IMPA, immune‐mediated polyarthritis; SRMA, steroid‐responsive meningitis arteritis.

**Supplementary Table 1.** Demographic characteristics of dogs with IMHA, ITP and IMPA.
**Supplementary Table 2.** Summary of diagnostic testing performed in dogs diagnosed with IMHA, ITP and IMPA; serology performed via SNAP 4Dx Plus (IDEXX Laboratories, Inc), Angiostrongylus vasorum antigen performed via AngioDetect rapid assay (IDEXX Laboratories, Inc).
**Supplementary Table 3.** Clinicopathological variables for dogs with IMHA and ITP.
**Supplementary Table 4.** Features of disease relapse among dogs with IMHA, ITP, and IMPA.
**Supplementary Table 5.** Characteristics of initial immunosuppressive treatment, treatment status at the time of relapse and discontinuation of treatment among dogs with IMHA, ITP, and IMPA.
