# Supplementary material for: Comparison of timing of relapse in dogs with nonassociative immune‐mediated hemolytic anemia, thrombocytopenia, or polyarthritis
Source: J Vet Intern Med. 2024 Feb 2;38(2):1035–42. doi: 10.1111/jvim.17004 (PMC10937512; doi:10.1111/jvim.17004)
Supplement: Supplementary file 1 — Supplementary Figure 1. Flow diagram demonstrating reasons for exclusion among 116 dogs identified with IMHA during the study period. *5 dogs presenting with relapse of historical IMHA included in the case‐control study comparing rates of relapse in dogs that were or were not vaccinated after initial diagnosis. IMHA, immune‐mediated hemolytic anemia. Supplementary Figure 2. Flow diagram demonstrating reasons for exclusion among 79 dogs identified with ITP during the study period. *2 dogs presenting with relapse of historical ITP included in the case‐control study comparing rates of relapse in dogs that were or were not vaccinated after initial diagnosis. ITP, immune thrombocytopenia; PLT, platelet concentration. Supplementary Figure 3. Flow diagram demonstrating reasons for exclusion among 57 dogs identified with IMPA during the study period. IMPA, immune‐mediated polyarthritis; SRMA, steroid‐responsive meningitis arteritis. [file JVIM-38-1035-s001.pdf]

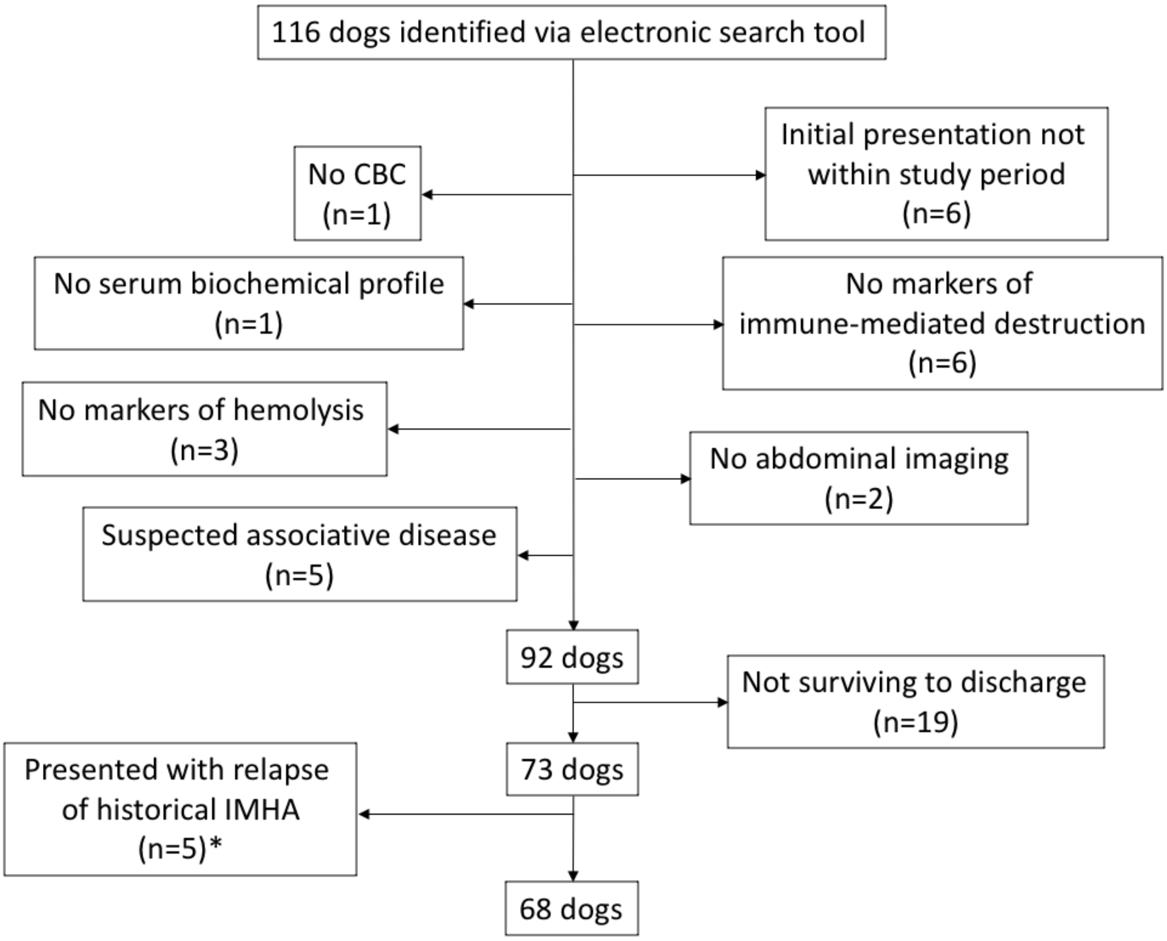

Supplementary Figure 1: Flow diagram demonstrating reasons for exclusion among 116 dogs identified with IMHA during the study period. *5 dogs presenting with relapse of historical IMHA included in the case-control study comparing rates of relapse in dogs that were or were not vaccinated after initial diagnosis. IMHA, immune-mediated hemolytic anemia.

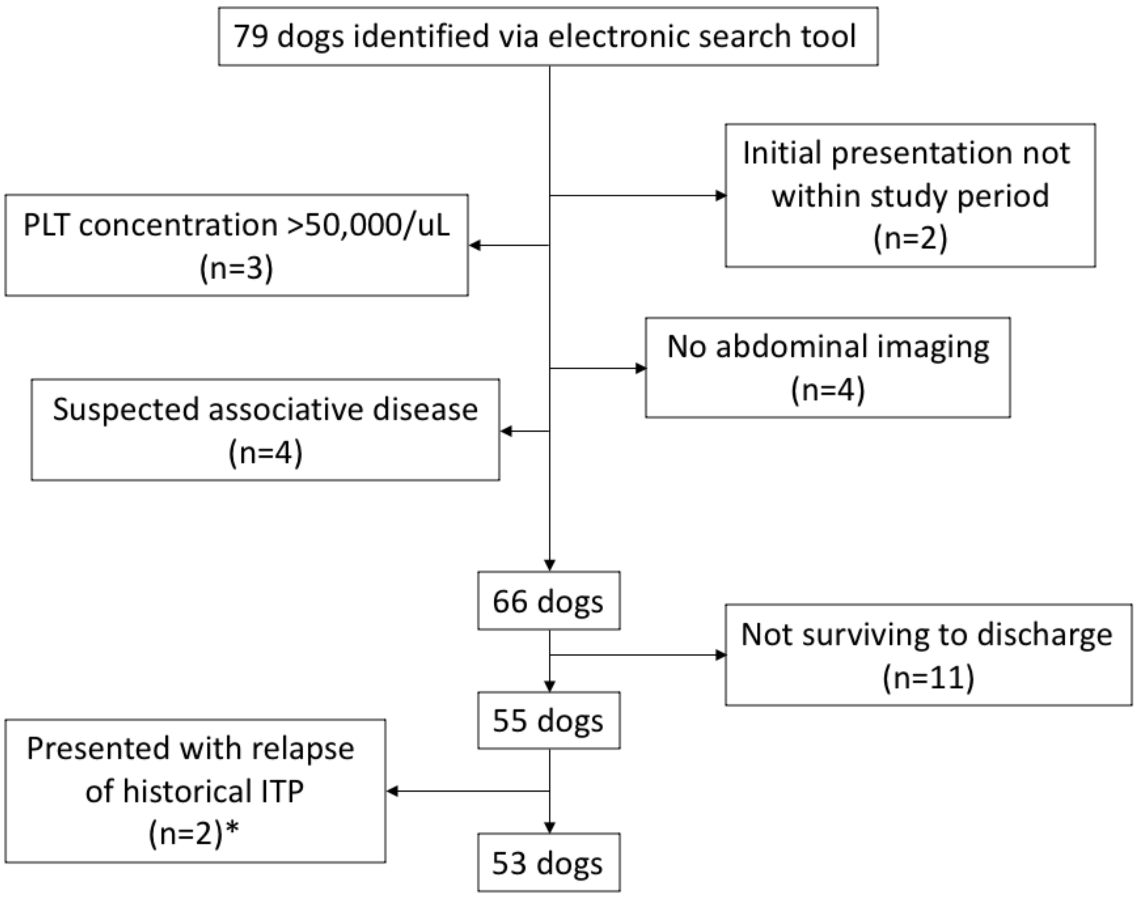

Supplementary Figure 2: Flow diagram demonstrating reasons for exclusion among 79 dogs identified with ITP during the study period. *2 dogs presenting with relapse of historical ITP included in the case-control study comparing rates of relapse in dogs that were or were not vaccinated after initial diagnosis. ITP immune thrombocytopenia, PLT platelet concentration.

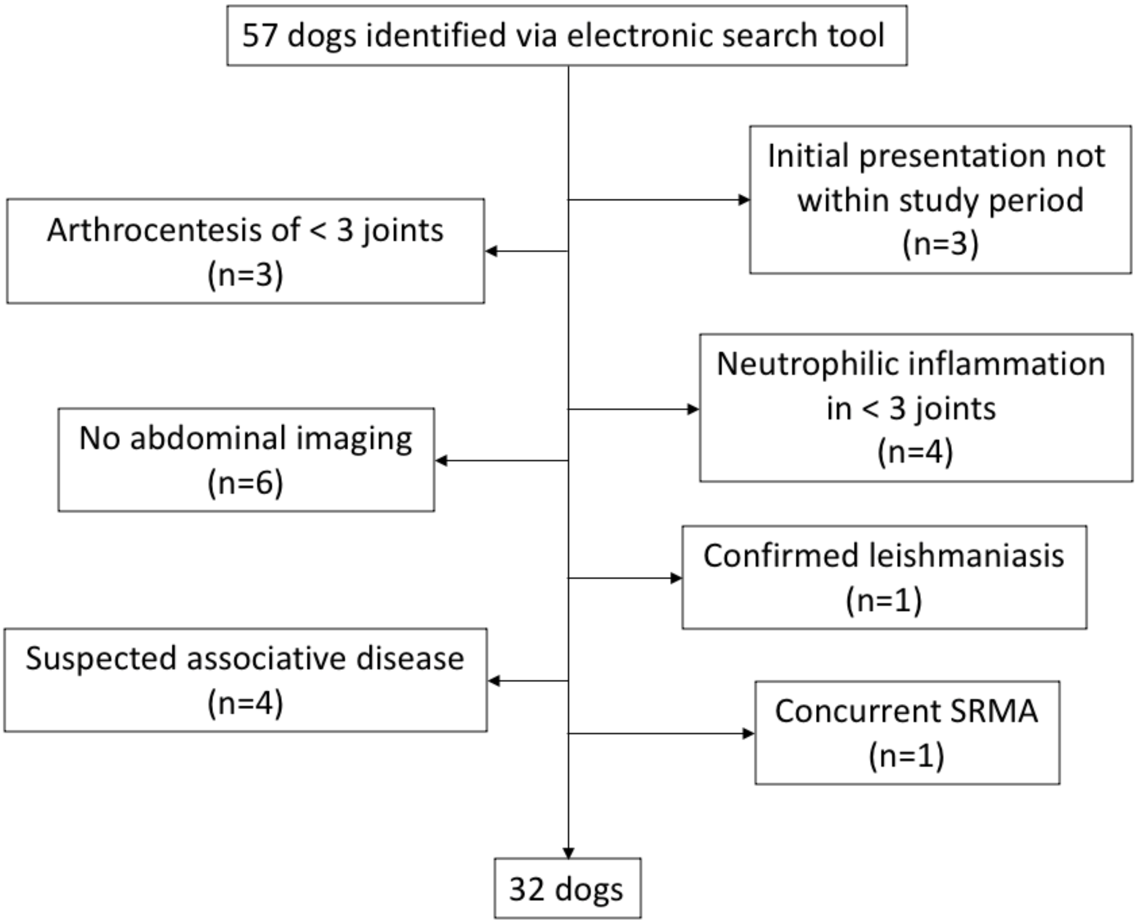

Supplementary Figure 3: Flow diagram demonstrating reasons for exclusion among 57 dogs identified with IMPA during the study period. IMPA immune-mediated polyarthritis, SRMA steroid-responsive meningitis arteritis.
