# Supplementary material for: Comparison of timing of relapse in dogs with nonassociative immune‐mediated hemolytic anemia, thrombocytopenia, or polyarthritis
Source: J Vet Intern Med. 2024 Feb 2;38(2):1035–42. doi: 10.1111/jvim.17004 (PMC10937512; doi:10.1111/jvim.17004)
Supplement: Supplementary file 2 — Supplementary Table 1. Demographic characteristics of dogs with IMHA, ITP and IMPA. Supplementary Table 2. Summary of diagnostic testing performed in dogs diagnosed with IMHA, ITP and IMPA; serology performed via SNAP 4Dx Plus (IDEXX Laboratories, Inc), Angiostrongylus vasorum antigen performed via AngioDetect rapid assay (IDEXX Laboratories, Inc). Supplementary Table 3. Clinicopathological variables for dogs with IMHA and ITP. Supplementary Table 4. Features of disease relapse among dogs with IMHA, ITP, and IMPA. Supplementary Table 5. Characteristics of initial immunosuppressive treatment, treatment status at the time of relapse and discontinuation of treatment among dogs with IMHA, ITP, and IMPA. [file JVIM-38-1035-s002.pdf]

Supplementary Table 1: Demographic characteristics of dogs with IMHA, ITP and IMPA.

		IMHA	ITP	IMPA
Dogs surviving to discharge		73	55	32
Age at presentation (years)		6.70 (SD ± 2.88)	6.61 (SD ± 2.61)	5.28 (SD ± 3.14)
Bodyweight (kg)		16.0 (IQR 8.9-25.0)	16.7 (IQR 11.4-26.4)	13.85 (IQR 9.8-28.4)
Gender	Female neutered (%)	35 (47.9)	31 (56.4)	13 (40.6)
	Male neutered (%)	24 (32.9)	13 (23.6)	10 (31.3)
	Female entire (%)	8 (11.0)	6 (10.9)	2 (6.3)
	Male entire (%)	6 (8.2)	5 (9.1)	7 (21.9)

IQR: Inter-quartile range; SD: standard deviation

Supplementary Table 2: Summary of diagnostic testing performed in dogs diagnosed with IMHA, ITP and IMPA; serology performed via SNAP® 4Dx® Plus (IDEXX Laboratories, Inc.), *Angiostrongylus vasorum* antigen performed via AngioDetect™ rapid assay (IDEXX Laboratories, Inc.)

Test performed	IMHA	ITP	IMPA
Serology (%)	67 (91.8)	49 (89.1)	29 (90.6)
Urinalysis (%)	68 (93.2)	41 (74.5)	32 (100)
Thoracic imaging (%)	70 (95.9)	54 (98.2)	31 (93.9)
Abdominal imaging (%)	73 (100)	55 (100)	32 (100)
Serum biochemistry (%)	73 (100)	55 (100)	32 (100)
Fecal Baermann's (%)	-	18 (32.7)	-
A. vasorum antigen (%)	-	33 (60.0)	-

Supplementary Table 3: Clinicopathological variables for dogs with IMHA and ITP.

Variable	IMHA	ITP	Reference range
Manual platelet concentration (/μL)	-	20,000 (IQR 5,000-20,000)	150,000 - 900,000
PCV (%)	14 (IQR 12-19)	36 (± 11.07)	37-55
Total protein (g/dL)	7.37 (± 0.76)	6.91 (± 1.27)	4.9-7.1
Urea (mg/dL)	21.0 (IQR 14.57-25.77)	7.0 (IQR 5.2-9.4)	8.4-25.5
Albumin (g/dL)	3.14 (IQR 2.83-3.52)	-	2.8-3.9
Total bilirubin (mg/dL)	0.79 (IQR 0.25-1.78)	-	0.0-0.14
Alanine aminotransferase activity (U/L)	37 (IQR 24-85)	-	13-88

Supplementary Table 4: Features of disease relapse among dogs with IMHA, ITP and IMPA.

Disease group	IMHA	ITP	IMPA
Dogs surviving to discharge	73	55	32
Dogs presenting with relapse of historical disease	5	2	0
Total dogs relapsing	9	8	10
Relapse <12 months (%)	5 (55.6)	4 (50)	9 (90)
Relapse 12-24 months (%)	2 (22.2)	3 (37.5)	0 (0)
Relapse >24 months (%)	2 (22.2)	1 (12.5)	1 (10)
12-month relapse rate (%)	11.4 (95% CI 3.7-26.5)	11.4 (95% CI 3.1-29.2)	34.6 (95% CI 15.8-65.7)
24-month relapse rate (%)	17.5 (95% CI 7.0-36.1)	22.6 (95% CI 9.1-46.5)	40.9 (95% CI 18.7-77.7)
Dogs having multiple relapses (%)	1 (11.1)	2 (25)	5 (50)
Time from diagnosis to relapse	6 (IQR 2.5-27.5)	12.0 (± 9.43)	6.79 (IQR 2.36-8.2)

Supplementary Table 5: Characteristics of initial immunosuppressive treatment, treatment status at the time of relapse and discontinuation of treatment among dogs with IMHA, ITP and IMPA.

	IMHA	ITP	IMPA
Median prednisolone dose at discharge (mg/kg/day)	2.20 (IQR 1.97-2.98)	2.19 (IQR 1.92-2.55)	2.42 (IQR 1.88-3.0)
Time from discharge to first prednisolone dose reduction (days)	22 (IQR 17.25-27.0)	28 (IQR 17.5-37.0)	20 (IQR 15.0-24.8)
Prednisolone dose at first dose reduction (mg/kg/day)	1.61 (IQR 1.47-2.04)	1.67 (IQR 1.5-1.90)	1.56 (IQR 1.41-1.92)
Time between first and second dose reduction (days)	21 (IQR 14.8-29.0)	24 (IQR 14.8-30.3)	21 (12.5-32.3).
Prednisolone dose at second dose reduction (mg/kg/day)	1.07 (IQR .96-1.30)	1.12 (IQR .91-1.30)	1.11 (IQR .95-1.32)
Total duration of treatment with prednisolone (months)	4.92 (IQR 3.93-6.78)	5.95 (IQR 4.42-8.37)	5.22 (IQR 3.3-7.16)
Total duration of treatment with second agent (months)	6.08 (IQR 5.21-7.75)	7.18 (IQR 4.99-8.58)	7.57 (IQR 3.3-13.6)
Dogs with >24 months follow up receiving second agent at discharge	34/40 (85.0%)	22/31 (71.0%)	13/22 (59.1%)
Proportion of relapsing dogs that received a second immunosuppressive drug at discharge	6/9 (66.7%)	6/8 (75.0%)	7/10 (70.0%)

Dogs that did not relapse receiving second agent at discharge	28/31 (90.3%)	16/24 (66.7%)	6/12 (50%)
Dogs that relapsed whilst receiving immunosuppressive treatment	6/9 (66.7%)	4/8 (50.0%)	4/10 (40%)
Dogs that relapsed after immunosuppressive treatment was discontinued	3/9 (33.3%)	4/8 (50%)	6/10 (60%)
Proportion of dogs in which immunosuppressive therapy was discontinued	54/71 (76.1%)	17/52 (32.7%)	20/29 (69.0%)
